# Efficacy of Palmitoylethanolamide and Luteolin Association on Post-Covid Olfactory Dysfunction: A Systematic Review and Meta-Analysis of Clinical Studies

**DOI:** 10.3390/biomedicines11082189

**Published:** 2023-08-03

**Authors:** Anna Paola Capra, Alessio Ardizzone, Lelio Crupi, Fabrizio Calapai, Michela Campolo, Salvatore Cuzzocrea, Emanuela Esposito

**Affiliations:** 1 Department of Chemical, Biological, Pharmaceutical and Environmental Sciences, University of Messina, 98166 Messina, Italy; annapaola.capra@unime.it (A.P.C.); aleardizzone@unime.it (A.A.); lelio.crupi@student.unime.it (L.C.); fabrizio.calapai@unime.it (F.C.); campolom@unime.it (M.C.); salvator@unime.it (S.C.); 2 Department of Clinical and Experimental Medicine, University of Messina, 98125 Messina, Italy

**Keywords:** palmitoylethanolamide (PEA), Luteolin, CoUltraPEALut, SARS-CoV-2, COVID-19, Post-Covid Olfactory Dysfunction (PCOD), respiratory disorders

## Abstract

Post-Covid Olfactory Dysfunction (PCOD) is characterized by olfactory abnormalities, hyposmia, and anosmia, which are among the most often enduring symptoms in individuals who have recovered from SARS-CoV-2 infection. This disorder has been reported to persist in subsets of patients well after 12 months following infection, significantly affecting their quality of life. Despite the high prevalence of PCOD among patients who suffered from SARS-CoV-2 infection, specific therapeutic strategies are still limited. Among these, emerging evidence seems to indicate the administration of CoUltraPEALut, a combination of micronized Palmitoylethanolamide (PEA), an endogenous fatty acid amide, and Luteolin, a natural antioxidant flavonoid, as a viable therapy, especially when given as an adjuvant to olfactory training. Based on the above, a systematic review and a meta-analysis of the literature were conducted, with the aim of evaluating the efficacy of CoUltraPEALut as an addition to olfactory training (OT), in treating PCOD symptoms. Pubmed (MEDLINE), Embase (OVID), and Web of Science scientific databases were screened from the inception until 31 May 2023, and a total of 407 articles were recovered; only five of these studies (441 total patients between treated and control groups) were included in the systematic review. CoUltraPEALut demonstrated significant efficacy in the overall recovery of the olfactory function, compared to the conventional therapy, suggesting that it could represent a possible future adjuvant treatment for PCOD.

## 1. Introduction

N-acetylethanolamines (NAEs) are a family of endogenous lipid molecules that include palmitoylethanolamide (PEA) [1,2].

PEA is produced “on demand” by our body, in response to stressful conditions or inflammatory stimuli, thus denoting its key role in maintaining cellular homeostasis [3]. Even though it is speculated that PEA can interact via binding to certain nuclear receptors like PPAR, and in particular to PPAR-α, as well as to a cannabinoid-type receptor GPR55, the mechanism of action of PEA is not yet completely understood [4]. Although PEA has been shown to have powerful protective activities by Autacoid Local Injury Antagonism (ALIA) mechanism [5], one of its main problems is its poor bioavailability [2]. Concerning this, pharmaceutical micronization processes have proven to be very advantageous for dissolution enhancement of poorly water-soluble drugs [6]. PEA-um, as a new formulation of PEA, has been shown to be effective in reducing inflammatory processes [2].

The conjugation of PEA with antioxidant molecules may increase its efficacy and provide stronger pharmacological effects. In fact, PEA lacks a direct antioxidant ability for preventing the generation of free radicals and mitigating the damage that free radicals cause to DNA, lipids, and proteins.

In recent years, scientific interest in flavonoids has increased enormously; in fact, considering their biological effects, these precious natural molecules can be a valuable support against several diseases, providing beneficial effects for human health. In particular, Luteolin occurs naturally in many vegetables and fruits, and its therapeutic effects on various pathologies were demonstrated by much scientific evidence. Due to its biological characteristics, it has potent anti-inflammatory, anti-diabetic, antioxidant, and anticancer effects, which improve patient clinical outcomes in many pathological settings.

The pharmaceutical formulation combining PEA with Luteolin, known as CoUltraPEALut, has been shown to have neuroprotective and neuroregenerative properties following traumatic brain injury (TBI) or MPTP-induced Parkinson’s disease [7]. 

These beneficial effects on human health would suggest the use of CoUltraPEALut also in different clinical pictures. 

Over the past three years, most clinical efforts have been directed toward the COVID-19 pandemic, which has constituted one of the worst global public health emergencies. 

Although the rapid development of vaccines has mitigated the impact of COVID-19 on severe clinical outcomes and mortality rate, the search for pharmacological strategies effective in moderating the long COVID-19 syndrome remains an unsolved clinical challenge. The most typical symptoms of post-COVID-19 syndrome include fatigue, headache, dyspnea, hoarseness of voice, and myalgia, and the presence of comorbidities may worsen long-term illnesses [8]. In this scenario, many COVID-19 patients exhibited olfactory dysfunction, now identified as Post-Covid Olfactory Dysfunction (PCOD), which is a major concern following infection [9]. Both peripheral and central mechanisms have been suggested as possibly involved in the occurrence of PCOD [10]. As far as a peripheral mechanism is concerned, SARS-CoV-2 is known to have a marked tropism for respiratory epithelial cells, particularly for sustentacular cells, because of the high expression rate on these cells of ACE-2 receptors [11]. On the other hand, there is evidence showing that SARS-CoV-2 has only marginal affinity for olfactory sensory neurons, namely the mediators of the olfactory perceptions [12]. However, it has been proposed that an extensive damage of the respiratory epithelium may result in long-term reduction of the olfactory functionality. Recent evidence seems to support this hypothesis, in that a primary damage of sustentacular cells may disrupt homeostasis of nearby olfactory sensory neurons, thus inducing a marked and sustained alteration in the gene expression of olfactory sensory neurons [13].

On the other hand, it has been suggested that several neurological signs in SARS-CoV-2 patients may be due to the diffusion of the infectious virion, or of inflammatory mediators, from the cribriform plate up to the olfactory bulb via paracellular or transcellular pathways [10]. However, only fragmentary information is presently available on the involvement of the olfactory bulbs during SARS-CoV-2 infection. Evidence from COVID-19 autopsy reports has been provided, showing marked inflammation in the olfactory bulbs, along with significantly increased SARS-CoV-2 RNA levels in the olfactory bulbs compared to other cerebral regions [14,15]. Furthermore, some studies, using MRI techniques, discovered morphological changes (mainly a decreased volume) in the olfactory bulb and in the related cortical areas [16,17].

Considering these assumptions, our review question focuses on whether CoUltraPEALut could improve olfactory loss following COVID-19 infection. 

Therefore, by investigating PubMed (MEDLINE), Embase (OVID), and Web of Science scientific databases, this systematic review and meta-analysis aimed to assess the efficacy of CoUltraPEALut in improving olfactory dysfunction-related outcomes in long COVID-19 patients.

## 2. Methods

### 2.1. Search Strategy

We performed the literature search by using PubMed (MEDLINE), Embase (OVID), and Web of Science bibliographic databases. We used the Preferred Reporting Items for Systematic Review and Meta-analysis Protocols (PRISMA-P) guidelines in order to report a detailed search strategy of the articles. Based on the qualifying requirements listed in Table 1 and taking into account only English-language literature, APC and AA conducted the bibliographic search.

The search strategy was developed, and the study was supervised by two content experts (MC and EE). 

We searched for a period comprising from 2020 to 2023, no geographic exclusion criteria were imposed. Terms related to CoUltraPEALut in the context of long COVID-19 were explored in PubMed (MEDLINE), Embase (OVID), and Web of Science databases by using specific keywords summarized in Table 2.

### 2.2. Study Selection

We first performed our search using PubMed (MEDLINE), Embase (OVID), and Web of Science databases and then we excluded duplicates. After this step, the titles and abstracts of all findings found were then separately examined by the two review authors (APC and AA) to weed out any records that were not pertinent. After that, we carefully examined the full-text articles to select those that met the requirements for eligibility. The involvement of a third review author (EE) helped to reconcile differing viewpoints.

The included studies’ data were extracted by two authors (APC and AA). We collected the following information from the five included studies: title, author(s), publication year, study catchment area (i.e., geographic zone), study participants, and associated clinical outcomes.

### 2.3. Assessment of Risk of Bias

Using the Cochrane Risk of Bias 2.0 (RoB2) tool, two reviewers (APC and AA) independently evaluated the quality of the eligible records. 

Specifically, they evaluated the risk of bias on five domains: randomization process (D1), deviations from the intended interventions (D2), missing outcome data (D3), measurement of the outcome (D4), and selection of the reported result (D5).

This assessment led to the classification of the study’s value as low, medium, or high. A third review author (EE), who assisted in attaining consensus, was brought in to help settle differences in score allocations. None of the papers were deemed to be at a high risk of bias after the authors’ evaluations.

### 2.4. Data Synthesis Methods for Meta-Analysis

For statistical analysis in the meta-analysis, we employed an odds ratio (OR) measure and the random-effects model with the Mantel–Haenszel approach. We successfully combined estimates of the variant effect (OR) and its corresponding 95% confidence interval (CI). The forest plots were graphically examined to determine the heterogeneity, which was then measured using the I^2^ statistic [18,19]. The meta-analysis of the pooled data was carried out using Review Manager (RevMan Version 5.4., The Nordic Cochrane Centre, The Cochrane Collaboration: Copenhagen, Denmark, 2014).

## 3. Results and Discussion

### 3.1. Findings from Systematic Search

**Figure 1:**
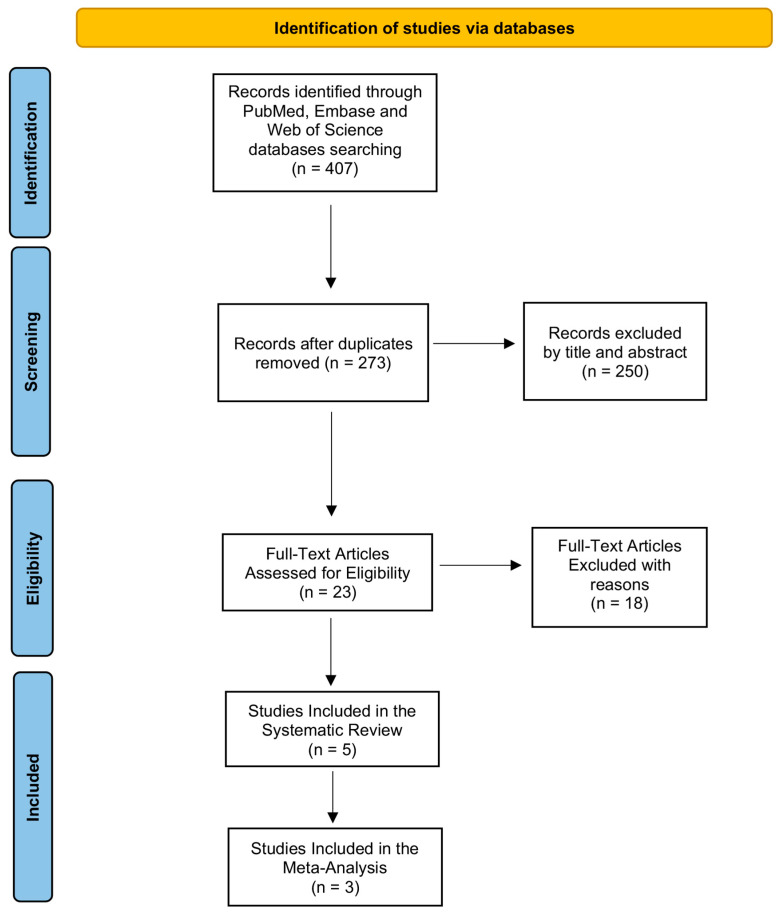
PRISMA flow diagram. The picture outlines each phase of the search strategy and screening procedure, which was carried out in accordance with PRISMA-P guidelines.

Using the PRISMA-P flowchart, we show the entire screening procedure in Figure 1. By combining the search terms listed in Table 2, we were able to find 407 records in the PubMed (MEDLINE), Embase (OVID), and Web of Science databases. After removing the duplicates, we had 273 records left, which we next examined for eligibility considering the title and abstract. Because their title and abstract were not pertinent to our review topic, we disregarded 250 publications in this phase. 

After checking 23 articles’ entire texts for eligibility, we eliminated 18 records since they did not meet the inclusion and exclusion requirements. As illustrated by the PRISMA Flowchart presented in Figure 1, we finally included five studies in our systematic review that assessed the effectiveness of CoUltraPEALut in PCOD. We also performed a meta-analysis to determine if CoUltraPEALut was successful in treating PCOD patients. 

Only three records that examined the effects of CoUltraPEALut on olfactory dysfunction (measured by Sniffin’ Sticks and reported as TDI score) compared to a control group were chosen for this purpose from the five studies that were included in the systematic review.

### 3.2. Evaluation of Included Studies in the Systematic Review

Olfactory dysfunction was evaluated in each included trial using the Sniffin’ Stick score (TDI score) between the control group and treatment group both at T0 (baseline) and T1 (endpoint). Overall, in each study (summarized in Table 3), CoUltraPEALut treatment plus olfactory training (OT) considerably increased the TDI score values compared to the control group improvement, indicating a stronger recovery of olfactory function.

Twelve people, ranging in age from 18 to 90, were included in the study of D’Ascanio et al. Patients had a documented history of COVID-19 and anosmia or hyposmia that persisted for at least 90 days following a negative COVID-19 nasopharyngeal swab result. Subjective reports at T0, the beginning of the trial, indicated persistent smell problems. The evaluation at T1 (30 days following the trial’s completion), which included OT sessions and adherence to the treatment group’s supplement regimen, was conducted on all study participants [20]. Treatment combining olfactory rehabilitation with oral PEA and luteolin supplementation has been associated with increased olfactory function recovery (evaluated by Sniffin’ Sticks), particularly pronounced in individuals with chronic olfactory impairment [20].

De Luca et al. included 69 patients with a verified history of COVID-19, including 43 women and 26 men, ages 18 to 80, with anosmia/hyposmia that persisted for at least 180 days (six months) following a negative COVID-19 nasopharyngeal swab. They were assigned to three groups: (1) individuals with prior OT received a daily PEA-LUT oral supplement and continued OT; (2) Training-Naïve 1 (PEA-LUT plus OT) patients consumed one sublingual sachet of PEA-LUT per day and performed OT three times a day; and (3) patients consumed one sublingual sachet of PEA-LUT per day and underwent no additional intervention [21]. 

Overall, the treatment using oral PEA-LUT and olfactory training improved olfactory dysfunction in individuals with protracted COVID and chronic olfactory loss [21] as evidenced by improvement in TDI score and an improved perception of smells through the administration of a questionnaire that contained 52 different odors.

With 185 patients, ranging in age from 18 to 80, who had verified COVID-19 histories and anosmia or hyposmia that persisted for more than 180 days (six months) after a later negative COVID-19 nasopharyngeal swab, Di Stadio et al. conducted a multicenter double-blinded randomized clinical study. These are the two study groups’ definitions: (2) Conventional therapy (control group): daily treatment with placebo and OT; (1) Intervention therapy (intervention group): daily treatment with PEA-LUT oral supplement [22].

In a different study, Di Stadio and colleagues recruited a different group of patients with the same long covid condition, aged 18 to 60 years with a confirmed history of COVID-19 and anosmia/hyposmia persisting ≥180 days after a negative COVID-19 nasopharyngeal swab. The COVID-19 group subjects were treated using um-PEA-LUT plus OT [23].

In addition, the most recent work of Stadio et al. evaluated once again the efficacy of CoUltraPEALut and OT compared to OT alone for the treatment of smell disorders in another Italian region, thus confirming in another set of analyses the advantages of PEA and Lut combination on the quality smell disorders in the post-COVID population [24]. The research comprised a total of 130 patients; 94 patients (49 women and 45 men, average age 36.7 ± 11.8) were placed in the treatment group, while 36 patients (21 women and 15 men, average age 50.5 ± 12.7) were placed in the control group. Patients in the therapy group had smell alteration lasting 8.8 ± 3.7 months on average, compared to 8.5 ± 2 months for patients in the control group [24]. Nevertheless, the authors cautioned that their findings on parosmia are constrained and that the difference in average ages between the groups has affected their interpretation of the data. This discovery was verified by the identification of age and sniffing score at T0 as components that influenced the parosmia resolution. In fact, the control group comprised younger patients than the treatment group, who might have benefited from spontaneous recovery, which is less likely in patients over 40. Therefore, it was noted that more research comparing patients of the same age is required to determine whether the PEA-Lut combination is effective in treating qualitative smell changes.

**Table 3:** Summary of the studies included in the systematic review that assessed the effectiveness of CoUltraPEALut for olfactory dysfunction.

First Author and Year of Publication	Number of Patients Included	CoUltraPEALut Dosage Regimen	Main Results	Reference
D’Ascanio et al., 2021	Total *n*: 12 patients with PCOD - Treatment group (OT + umPEA-LUT), *n*: 7 - Control group (OT + Placebo), *n*: 5	umPEA-LUT (PEA 700 mg and Luteolin 70 mg) once a day, for 30 consecutive days	Patients which received umPEA-LUT in combination to OT had a significant improvement in TDI scores, evaluated with the Sniffin’ Sticks identification test, compared to the Control group.	[20]
De Luca et al., 2022	Total *n*: 69 patients with PCOD - Previously trained group: individuals previously exposed to OT (OT + umPEA-LUT), *n*: 10 - Training-Naïve 1: individuals not previously exposed to OT (OT + umPEA-LUT) *n*: 43 - Training-Naïve 2: individuals not previously exposed to OT (umPEA-LUT without OT), *n*: 16	umPEA-LUT (PEA 700 mg and Luteolin 70 mg) once a day, for 90 consecutive days	Treatment with umPEA-LUT was associated with an improvement in PCOD and mental clouding symptoms. The effects were more pronounced when combining PEA-LUT and OT.	[21]
Di Stadio et al., 2022	Total *n*: 185 patients with PCOD - Treatment group (OT + umPEA-LUT), *n*: 130 - Control group (OT + Placebo), *n*: 55	umPEA-LUT (PEA 700 mg and Luteolin 70 mg) once a day, for 90 consecutive days	Patients receiving umPEA-LUT showed a significantly greater improvement in TDI scores in comparison to patients in the control group.	[22]
Di Stadio et al., 2023	Total *n*: 130 patients with PCOD reporting parosmia as a symptom - Treatment group (OT + umPEA-LUT), *n*: 94 - Control group (OT + Placebo), *n*: 36	umPEA-LUT (PEA 700 mg and Luteolin 70 mg) once a day, for 90 consecutive days	umPEA-LUT in combination with OT shows significant efficacy compared to OT alone, in treating quantitative olfactory alterations, measured as TDI scores. No significant effects on qualitative alterations (parosmia) were observed between the groups.	[24]
Di Stadio et al., 2023	Total *n*: 45 with PCOD - PCOD group (OT + umPEA-LUT), *n*: 45	(umPEA-LUT) PEA 700 mg and Luteolin 70 mg once a day, for 90 consecutive days	PCOD patients at the end of the umPEA-LUT treatment period showed significantly higher TDI scores compared to the baseline scores.	[23]

### 3.3. Meta-Analysis

The inclusion criteria (quantitative comparison of TDI score between treated patients and controls) were only met by three records from the screening method used to identify trials eligible for meta-analysis. 

Although there was significant heterogeneity (I^2^ = 89%), the total OR was 3.07 (95% CI: 2.22–3.92) and the test for overall effect was *p* < 0.00001.

**Figure 2:**
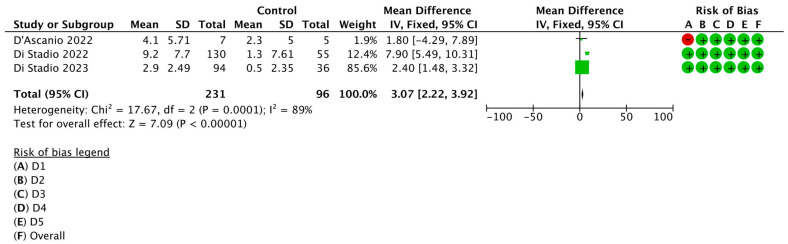
Forest plot of the studies analyzed in the quantitative synthesis [20,22,24]. By subtracting the value at endpoint with the value at baseline and calculating the combined SD, the forest plot shows how the TDI score improved in treated patients and control individuals. The impact estimate (ORs) is displayed as squares, with the size of each green square reflecting the weight assigned to each research in the meta-analysis. The 95% confidence intervals (CIs) for each effect estimate are shown as horizontal lines. The black diamond’s width, which indicates the total 95% CI, represents the overall effect of intervention. A measurement of heterogeneity is the I^2^ statistic. Effect size OR: 3.07 [2.22, 3.92]; *p* < 0.00001.

In addition, as can be seen in the forest plot (Figure 2), the ORs of each single study vary from 1.80 to 7.90. This outcome had a strong statistical significance, showing the significant increased rate of olfactory recovery after CoUltraPEALut administration associated with OT, compared to control subjected only to OT. 

**Figure 3:**
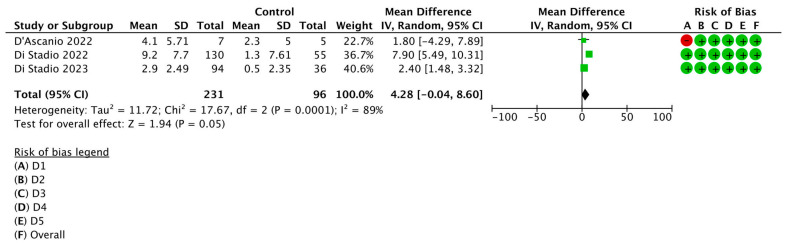
Forest plot of the studies analyzed in the quantitative synthesis [20,22,24]. By subtracting the value at endpoint with the value at baseline and calculating the combined SD, the forest plot shows how the TDI score improved in treated patients and control individuals. The impact estimate (ORs) is displayed as squares, with the size of each green square reflecting the weight assigned to each research in the meta-analysis. The 95% confidence intervals (CIs) for each effect estimate are shown as horizontal lines. The black diamond’s width, which indicates the total 95% CI, represents the overall effect of intervention. A measurement of heterogeneity is the I^2^ statistic. Effect size OR: 4.28 [−0.04, 8.60]; *p* = 0.05.

Furthermore, to emphasize how different statistical approaches impact the outcome, we choose to present the same data using the random-effect model due to the significant degree of heterogeneity detected in the previous analysis (Figure 3).

The employment of the random-effect model revealed the same degree of heterogeneity (I^2^ = 89%), while the total OR was greater, reaching a value of 4.28 although with an extended 95% CI: −0.04, 8.60 while the test for overall effect was less significant showing a *p* = 0.05. 

### 3.4. Discussion

Olfactory impairment or loss is one of the most common chemosensory dysfunctions associated with COVID-19 infection [25,26]. Its prevalence during the acute phase of the disease varies considerably among series, according to whether any degree of smell impairment is considered (i.e., hyposmia or only true anosmia), olfactory impairment is selectively evaluated, and not least the method used for olfactory loss detection [27]. Studies on the olfactory system show that SARS-CoV-2 can spread to the olfactory bulb and other parts of the central nervous system after entering the olfactory neuroepithelium [28]. SARS-CoV-2 is capable of persisting in patients’ olfactory bulbs even after they have recovered from an acute infection, leading to persistent olfactory impairments [29].

According to research, brain inflammation may be a frequent mediator of symptoms in patients with altered smell, who may also experience headache or brain fog [30,31]. Additionally, in the long COVID-19 research field, studies using MRI have shown that SARS-CoV-2 infection caused inflammatory changes to the olfactory bulbs [16].

Here, we focused on the improvement of clinical outcomes in PCOD patients following the administration of CoUltraPEALut, through a systematic search of different scientific databases, with the aim of investigating its beneficial effects in long COVID-19 patients.

By lessening the degree of neuroinflammation brought on by SARS-CoV-2, CoUltraPEALut treatment could enhance regeneration during olfactory training [32].

In fact, it was widely recognized that PEA has been shown to shift microglia’s polarization to a protective M2 phenotype, boosting brain regeneration and potentially favoring smell restoration [33]. Likewise, by preventing pro-inflammatory microglia from polarizing, luteolin prevents the deterioration of brain cells [34]. 

In accordance, our review including five studies and a total of 441 subjects (matching treated and control groups) showed persisting abnormalities of their sense of smell (hyposmia or anosmia) post COVID-19 infection. To solve olfactory dysfunction, in all these recent studies, including clinical trials and longitudinal study, the treatments with CoUltraPEALut, OT alone, or their combination, were compared. 

In every systematically searched clinical study, olfactory recovery was better when oral CoUltraPEALut supplementation was paired with OT than OT alone. In particular, the goal of this multimodal strategy was to lessen neuroinflammation in the olfactory system and foster a regenerative environment that could promote olfactory healing.

Taken as a whole, these clinical trials imply that CoUltraPEALut should be taken for at least 30 days to demonstrate statistically meaningful benefit, and extended follow-ups of at least 60–90 days seem to show even greater consistency.

Moreover, the combination of three studies in meta-analysis resulted in a total of 327 patients. The meta-analysis carried out statically confirmed the significant increase in olfactory recovery in CoUltraPEALut plus OT treated patients compared to OT patients (OR: 3.07; 95% CI: 2.22–3.92; test for overall effect *p* < 0.00001). Even if with a less statical significance when the random-effect model was employed (OR: 4.28; 95% CI: −0.04, 8.60; test for overall effect *p* = 0.05).

Our findings are consistent with current research trends in the PCOD field. Indeed, the combination of PEA-LUT and OT was noted in a 2022 Cochrane review on treatments for smell disorders caused by SARS-CoV-2 infection [35], although no statistical validation was performed.

Nevertheless, despite the high PCOD prevalence, therapeutic chances are presently limited. As stated, the most employed therapeutic approach is OT, a non-pharmacological procedure based on the repeated exposure of the affected subject to known odorants, usually twice a day for 12 weeks [36]. This approach proved effective in reverting olfactory dysfunctions consequent to viral infections and, more recently, in COVID-19 infection [37,38]. In this regard, a trial conducted by Denis et al. on 548 patients showed how OT, conducted for a mean period of 27.7 days, induced a significant improvement in 64.2% of patients (352/548) [39]. Unfortunately, there are still existing limitations to the use OT for the therapy of PCOD, the main ones being the high patient compliance needed and the length of the training periods needed in order to produce significant therapeutic results. 

Therefore, combined approaches of OT and pharmacological treatments have been proposed. To date, only a limited number of randomized clinical trials have demonstrated promising therapeutic strategies. This lack of specific medications for the treatment of PCOD likely reflects the gaps in the knowledge concerning the pathogenesis of long-term olfactory damage in COVID-19 patients.

Both oral and intranasal corticosteroids have been among the first substances tested as a potential therapy for PCOD, with some evidence, to date, supporting their efficacy.

As an example, a pilot study by Le Bon et al. showed that OT in combination with short-term oral steroids (10 days) was associated with a greater improvement in the olfactory score than OT alone [40]. Different molecules have been included as potential candidates in PCOD therapeutic regimen like Omega-3 supplements, topical Vitamin A, Alpha-lipoic acid, and theophylline, among others; however, evidence available is mostly limited and from non-randomised studies [41]. 

Thus, among the potential compounds studied, CoUltraPEALut is one of the few that has demonstrated promising efficacy.

To date, based on the most recent estimates of the World Health Organization (WHO) on the global spread of the COVID-19 pandemic, the above rates translate into tens of millions of subjects who either experienced or are presently experiencing different degrees of smell impairments and related quality of life deterioration [42,43]. 

In particular, PCOD has been reported at 12–24 months after COVID-19 remission in 3–25% of patients initially complaining of olfactory symptoms [44], thus constituting a real clinical challenge. As stated, from a clinical perspective, patients affected from PCOD complain of both quantitative (hyposmia and anosmia) and qualitative olfactory alterations. The latter includes parosmia, i.e., a distortion in smell perception, and phantosmia, i.e., olfactory hallucinations, among the main ones. 

In addition, it is important to underline that olfactory disorders may cause several daily life problems, such as altered social relations, decreased capacity for danger avoidance, abnormalities in food intake (either increased or decreased), and reduced working efficiency [45]. Moreover, chemosensory dysfunctions have been reported to be related to higher rates of depression and mood disorders [45]. 

Therefore, the validation of CoUltraPEALut in large-scale studies, as well as the future search of new care options represent an essential need to facilitate the full recovery of PCOD patients.

Despite that our work collected all the evidence published to date about the topic, several limitations persist. The small sample size, which is subject to underpowered analysis, is the main drawback. In addition, the baseline features of individuals can also vary, with noticeable variations in the degree and length of olfactory impairment at baseline. Additionally, we did not take into consideration co-factors like smoking history, other comorbidities like hypertension, cardiovascular diseases, obesity, diabetes, thyroid disorders, allergy, and psychiatric conditions, that can have a negative impact on the health condition and indirectly affect the physiological function of nasal mucosa. Furthermore, the pharmacological effect of CoUltraPEALut when administered in the presence of more complex clinical pictures that include chronic conditions and related symptoms together with PCOD is not known.

Moreover, the recovery of olfactory functions may require more time in the treatment group, despite improvements, and it is unclear whether adherence to the regimen and recovery would be sustained with a longer course of therapy due to the study’s limited follow-up, which ranges from 30 to 90 days.

Finally, the absence of placebo control can determine a different interpretation of conclusive findings and the reliability and validity of olfactory assessments can affect results. 

Furthermore, the five studies included analyzed the efficacy of CoUltraPEALut in different Italian populations. The data, therefore, related to individuals who are probably of Italian nationality, remain a contributing factor limiting the geographical heterogeneity. 

More research in larger populations is required to corroborate our early findings, determining the best time and dose regimes, and assessing the potential anti-inflammatory impact of CoUltraPEALut studying its pharmacokinetics, or its synergic activity with other therapies.

## 4. Conclusions and Future Perspectives

Overall, this systematic review and meta-analysis highlighted the significant benefits on olfactory dysfunction from COVID-19 following the administration of CoUltraPEALut, which are particularly advantageous in increasing the Sniffin’ Sticks test score. As far as we know, this is the first meta-analysis that probed the efficacy of CoUltraPEALut in long COVID-19 symptoms. However, despite the promising evidence, it is important to underline that there is a need for further large-scale clinical trials to further confirm these positive outcomes, and to better understand the mechanism of action of CoUltraPEALut underlying olfactory recovery.

Certainly, future well-designed clinical trials will be able to answer these questions, providing more details regarding the possible action of CoUltraPEALut on peripheral and central mechanisms involved in the occurrence of PCOD as well as on the modulation of inflammatory or neuroinflammatory pathways impacting olfactory bulbs. The collected data will be able to support not only the use of CoUltraPEALut in this peculiar picture of persistent symptoms linked to the previous SARS-CoV-2 infection but also in different clinical settings.

## Figures and Tables

**Table 1:** Description of inclusion and exclusion criteria employed for the literature search.

Inclusion Criteria	Exclusion Criteria
Clinical Trials or Randomized Controlled Trials evaluating CoUltraPEALut formulation (Glialia^®^, Epitech Group SpA, Saccolongo, Italy) in long COVID-19 olfactory dysfuction.	Observational studies, case-control studies, case reports, cross-sectional studies, cohort studies, editorials, letters, reviews, guidelines, abstracts and paper conferences, systematic reviews and meta-analyses, and ongoing studies. Articles not written in English.

**Table 2:** Keyword combinations used during the search strategy.

CoUltra PEALut	Long COVID-19
Palmitoylethanolamide, PEA, Luteolin, CoUltra PEALut, Co-Ultra PEALut, um-PEA-LUT, PEA-LUT, Glialia.	COVID-19, COVID19, COVID-19 Virus, COVID-19 Viruses, COVID-2019, SARS-CoV-2, SARS-CoV-2 Infection, Coronavirus, Coronaviruses, long COVID-19, long COVID-19 syndrome, long COVID-19 syndromes, post COVID-19, post COVID-19 syndrome, post COVID-19 syndromes, COVID-19 syndrome, COVID-19 syndromes, COVID-19 olfactory loss, COVID-19 olfactory dysfunction, COVID-19 olfaction, long-haul COVID syndrome, post-acute sequelae of SARS-CoV-2 infection (PASC).

## Data Availability

Data of this study are available to the corresponding author’s address.
